# Supplementary material for: Aerobic exercise reverses aging-induced depth-dependent decline in cerebral microcirculation
Source: bioRxiv. 2023 Feb 13:2023.02.12.528244. Preprint. [Version 1] doi: 10.1101/2023.02.12.528244 (PMC9949059; doi:10.1101/2023.02.12.528244)
Supplement: Supplement 1 [file NIHPP2023.02.12.528244v1-supplement-1.pdf]

bioRxiv preprint doi: https://doi.org/10.1101/2023.02.12.528244; this version posted February 14, 2023. The copyright holder for this preprint (which was not certified by peer review) is the author/funder, who has granted bioRxiv a license to display the preprint in perpetuity. It is made available under aCC-BY 4.0 International license.

[Source Files]

Figure3_SourceData1

Capillary RBC flux measured in young sedentary mice

Figure3_Source Data2

Capillary RBC flux measured in aged mice

Figure3_Source Data3

Venous flow measured in aged mice

Figure4_Source Data1

Capillary Po2 measured in aged mice

Figure4_Source Data2

Blood hematocrit level

Figure4_Source Data3

Arterial (and venous) Po2 measured in aged mice

Figure5_Source Data1

Peak hemodynamic response amplitude measured in aged mice

Figure6_Source Data1

Cortical capillary segment/length density measured in aged mice

Figure7_Source Data1

Behavioral scores from NORT and Y-maze test

bioRxiv preprint doi: https://doi.org/10.1101/2023.02.12.528244; this version posted February 14, 2023. The copyright holder for this preprint (which was not certified by peer review) is the author/funder, who has granted bioRxiv a license to display the preprint in perpetuity. It is made available under aCC-BY 4.0 International license.

[Supplementary Figures]

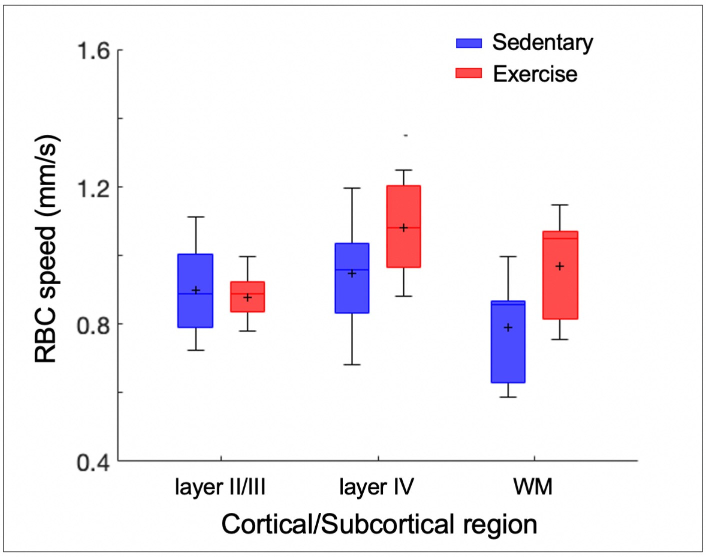

[Figure 3 – Figure supplement 1] Exercise induced alterations in capillary RBC speed. Capillary RBC speed across cortical layers II/III and IV, and subcortical white matter in aged sedentary and running mice, respectively. The analysis was made with 921, 486, and 112 capillaries across n=7 mice in the sedentary group and 1046, 465, and 238 capillaries across n=8 mice in the exercise group in cortical layers, II/III, IV, and the white matter. Statistical comparisons were carried out using Two-way ANOVA with Tukey post hoc test.

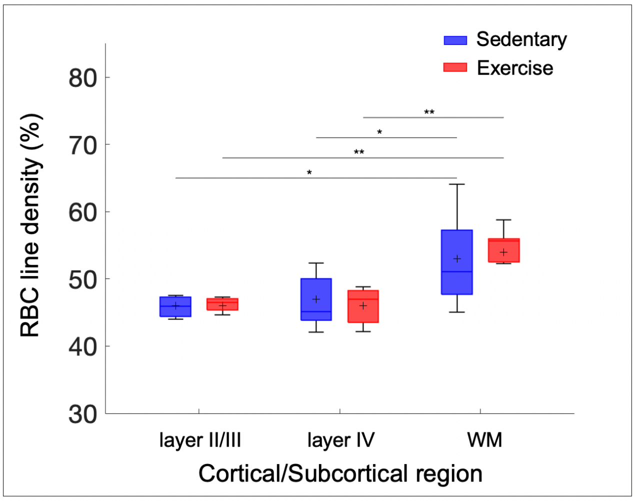

[Figure 3 – Figure supplement 2] Exercise induced alterations in capillary RBC line-density. Capillary RBC line-density across cortical layers II/III and IV, and subcortical white matter in aged sedentary and running mice. The analysis was made with 921, 486, and 112 capillaries across n=7 mice in sedentary group and 1046, 465, and 238 capillaries across n=8 mice in the exercise group in cortical layers, II/III, IV, and the white matter. Statistical

bioRxiv preprint doi: https://doi.org/10.1101/2023.02.12.528244; this version posted February 14, 2023. The copyright holder for this preprint (which was not certified by peer review) is the author/funder, who has granted bioRxiv a license to display the preprint in perpetuity. It is made available under aCC-BY 4.0 International license.

comparisons were carried out using Two-way ANOVA with Tukey post hoc test. The single-asterisk symbol (*) indicates p<0.05; the double-asterisk symbol (**) indicates p<0.01.

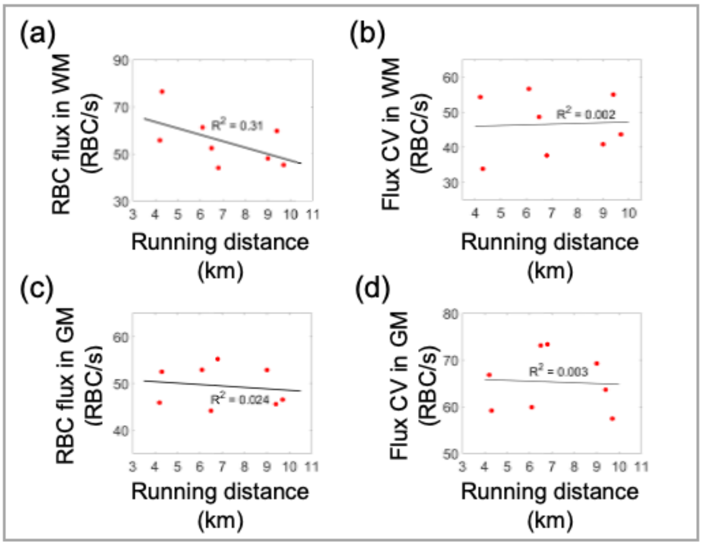

[Figure 3 – Figure supplement 3] Correlations between the capillary RBC flux and CV, and the running activity. Each data point represents an individual animal. The black solid line is the best linear regression fit. (a) and (b) Correlations between the capillary RBC flux and CV in the white matter and the average daily running distance, respectively. (c) and (d) Correlations between the CV of capillary RBC flux and CV in the gray matter and the average daily running distance, respectively. For each animal, the gray matter capillary RBC flux was calculated by averaging the acquired flux values from cortical layers II/III and IV.

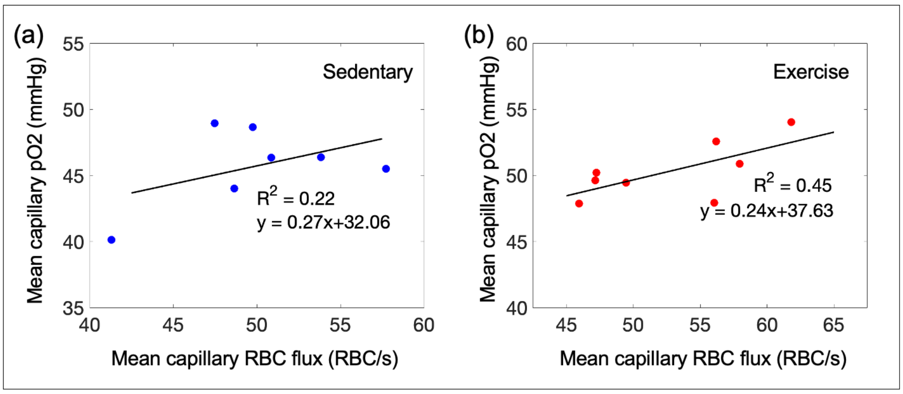

[Figure 4 – Figure supplement 1] Capillary mean-pO_2 vs capillary RBC flux in the mouse cortex. (a) and (b) Correlations between the mean capillary pO_2 and the mean capillary RBC flux in sedentary and running mice, respectively. The black solid line is the best fit result of each linear regression ($R^2 = 0.22, y = 0.27x + 32.06$, for sedentary mice and $R^2 = 0.45, y = 0.24x + 37.63$, for running mice). Each data point represents an

bioRxiv preprint doi: https://doi.org/10.1101/2023.02.12.528244; this version posted February 14, 2023. The copyright holder for this preprint (which was not certified by peer review) is the author/funder, who has granted bioRxiv a license to display the preprint in perpetuity. It is made available under aCC-BY 4.0 International license.

individual animal. For each animal, the mean capillary RBC flux and the mean capillary pO_2 were calculated by averaging the acquired RBC flux and pO_2 values from cortical layers II/III and IV, and cortical layers I, II/III, and IV, respectively. The correlation coefficient (the R value) for each group was converted to Fisher z value to compare difference between correlation coefficients of sedentary and running mice, and no significant difference was found (the observed z value = 0.44).

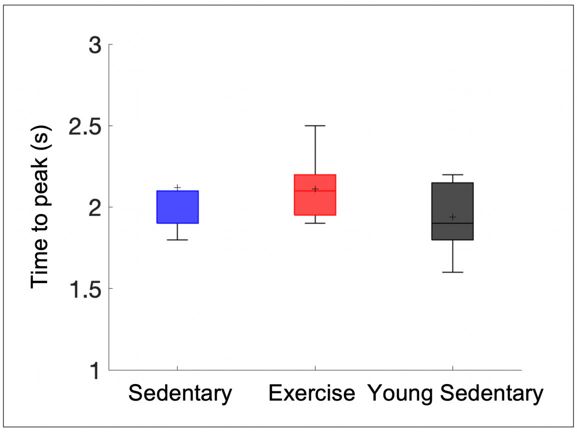

[Figure 5 – Figure supplement 1 Differences in the latency (time to peak) of stimulus-induced hemodynamic response between sedentary, running, and younger mice. Statistical comparisons were carried out using One-way ANOVA with Tukey post hoc test.

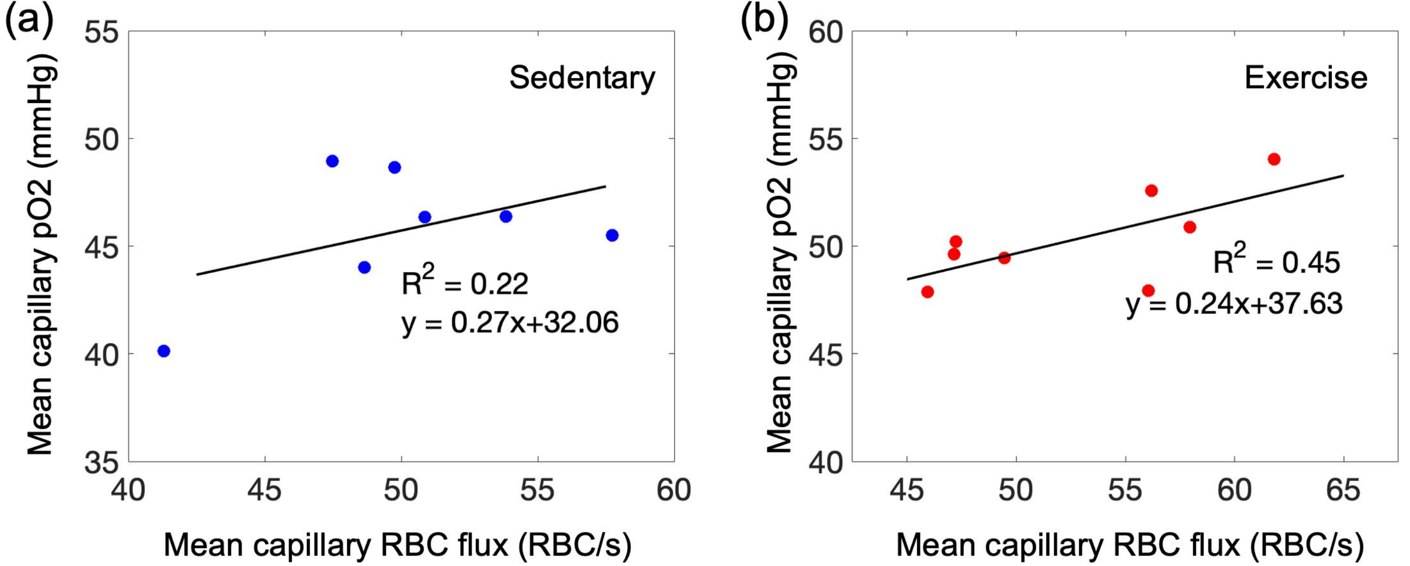
(a)
Sedentary
R^2 = 0.22
y = 0.27x+32.06
Mean capillary pO2 (mmHg)
Mean capillary RBC flux (RBC/s)
55
50
45
40
35
40
45
50
55
60
(b)
Exercise
R^2 = 0.45
y = 0.24x+37.63
Mean capillary pO2 (mmHg)
Mean capillary RBC flux (RBC/s)
60
55
50
45
40
45
50
55
60
65

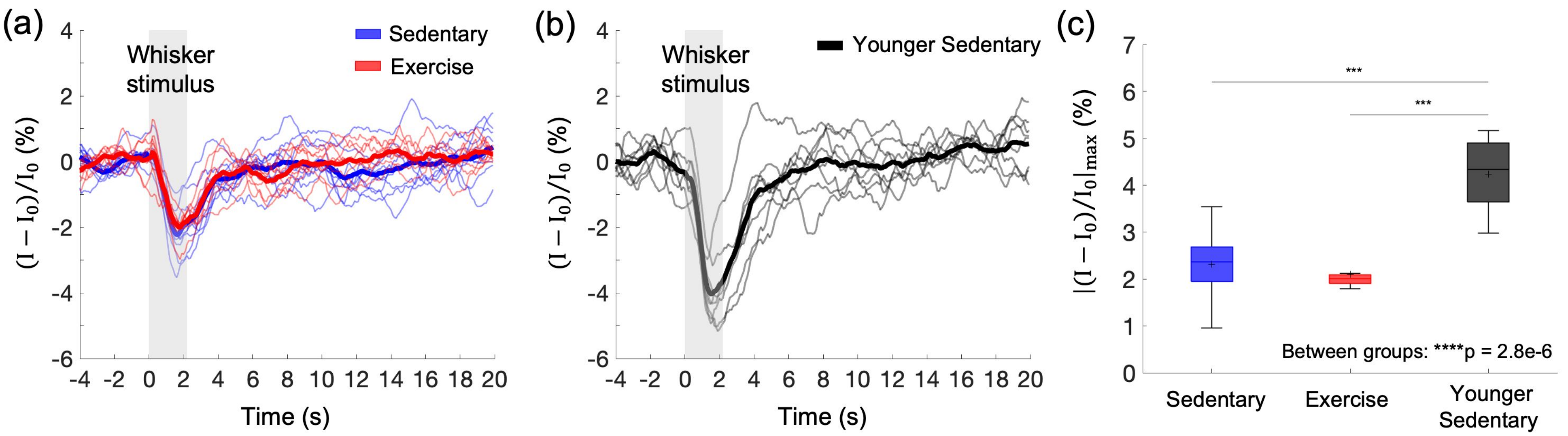

(a)
Whisker stimulus
Sedentary
Exercise
$(I - I_0)/I_0$ (%)
Time (s)
(b)
Whisker stimulus
Younger Sedentary
$(I - I_0)/I_0$ (%)
Time (s)
(c)
$|(I - I_0)/I_0|_{max}$ (%)
***
***
Between groups: ****p = 2.8e-6
Sedentary
Exercise
Younger Sedentary

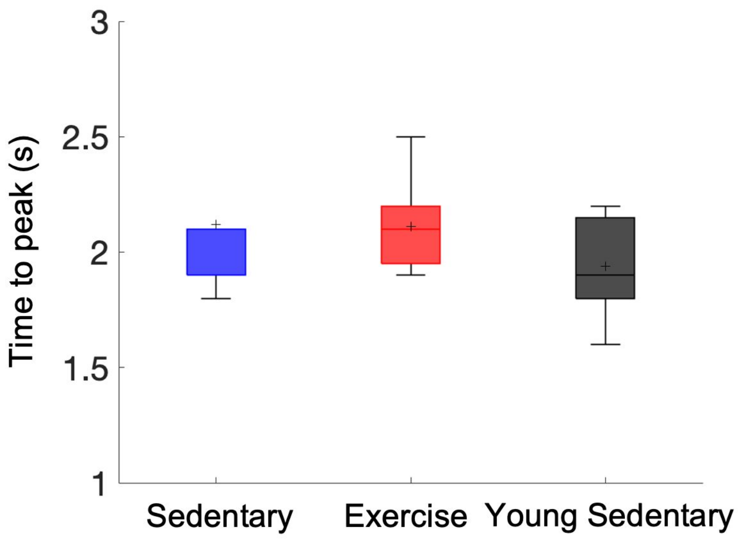

3
2.5
2
1.5
1
Time to peak (s)
Sedentary
Exercise
Young Sedentary

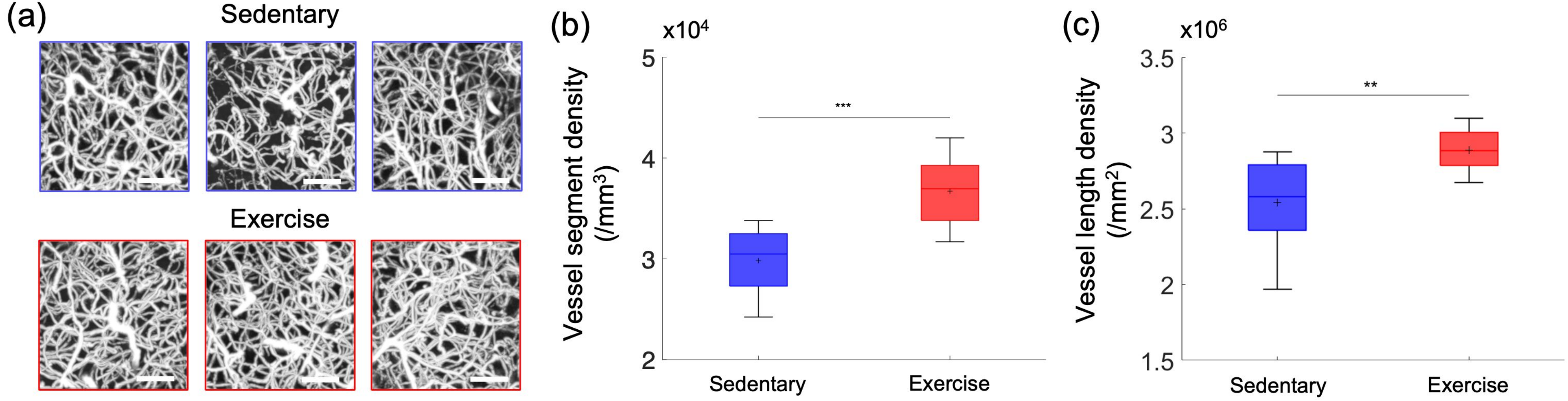

(a)
Sedentary
Exercise
(b)
x10^4
Vessel segment density (/mm^3)
5
4
3
2
***
Sedentary
Exercise
(c)
x10^6
Vessel length density (/mm^2)
3.5
3
2.5
2
1.5
**
Sedentary
Exercise

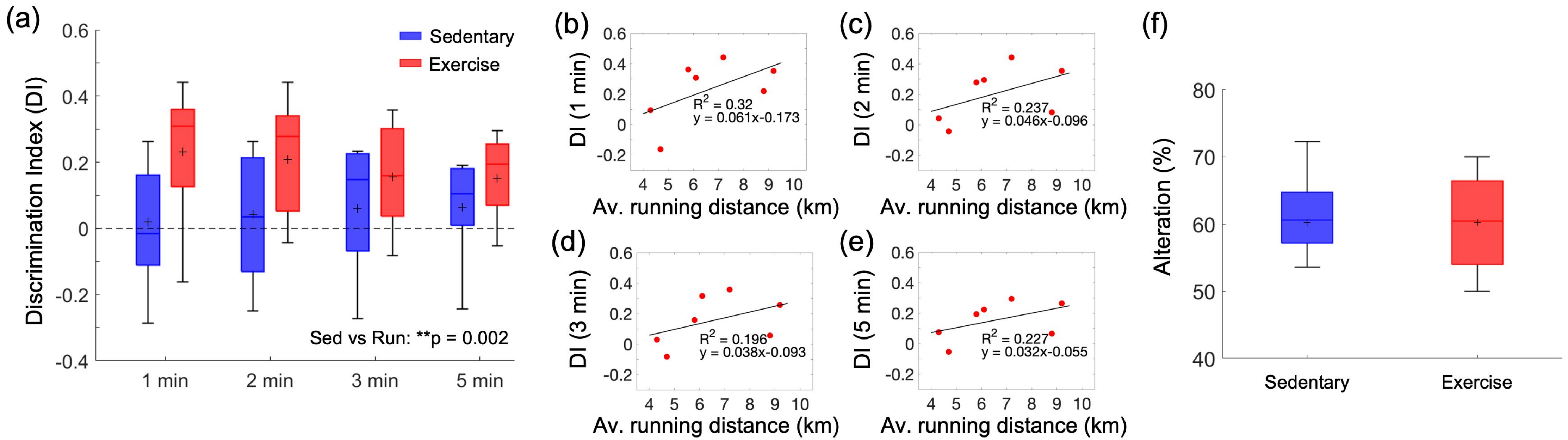

(a)
Sedentary
Exercise
Discrimination Index (DI)
0.6
0.4
0.2
0
-0.2
-0.4
1 min
2 min
3 min
5 min
Sed vs Run: **p = 0.002
(b)
DI (1 min)
R2 = 0.32
y = 0.061x-0.173
Av. running distance (km)
(c)
DI (2 min)
R2 = 0.237
y = 0.046x-0.096
Av. running distance (km)
(d)
DI (3 min)
R2 = 0.196
y = 0.038x-0.093
Av. running distance (km)
(e)
DI (5 min)
R2 = 0.227
y = 0.032x-0.055
Av. running distance (km)
(f)
Alteration (%)
80
70
60
50
40
Sedentary
Exercise
